# Self-perceived relations between artistic creativity and mental illness: a study into lived experiences

**DOI:** 10.3389/fpubh.2024.1353757

**Published:** 2024-06-11

**Authors:** Jenny Boumans, Arko Oderwald, Hans Kroon

**Affiliations:** ^1^Department of Mental Health Care and Participation, Trimbos Institute, Utrecht, Netherlands; ^2^Department of General Practice, Amsterdam University Medical Center, Amsterdam, Netherlands; ^3^Tranzo Scientific Center for Care and Wellbeing, Department of Social and Behavioral Sciences, Tilburg University, Tilburg, Netherlands

**Keywords:** mental illness, creativity, art, lived experiences, qualitative research, interpretative analysis

## Abstract

**Aim:**

To explore the self-perceived relationships between experiences of creativity and mental illness and to understand the meanings behind these relationships.

**Background:**

The idea that mental illness and artistic creativity are somehow related dates back to ancient times. There is some evidence for an actual correlation, but many questions remain unanswered on the nature and direction of the relationship. Qualitative contributions to the debate are scarce, and mainly focus on the potential benefits of participation in the arts for people with mental illness.

**Design:**

An explorative, interpretive study.

**Methods:**

Twenty-four professional and semi-professional artists with self-reported experience with mental illness, were recruited purposively. Unstructured in-depth interviews were conducted and transcripts were subjected to interpretive analysis, guided by a hermeneutic phenomenological frame.

**Results:**

Participants experience a range of interactions between artistic creativity and mental illness. Three constitutive patterns describe what these interactions look like: “flow as a powerful force”; “ambiguous self-manifestation”; and “narrating experiences of suffering.”

**Conclusion:**

The findings show that both the concept of creativity and the concept of mental illness, as well as their interrelationships, are layered and complex phenomena that can take on different meanings in people’s lives. The findings provide starting points for further research that goes beyond the polarized academic debate. Understanding the experiences of artists with mental illness can help shape the role of art in public mental health and mental health care.

## Introduction

The idea that mental illness and artistic creativity may be linked together dates back to ancient times, but to date no academic consensus has been reached on this topic. Epidemiological, population-based studies suggest that people with bipolar disorder and their family members are over-represented in creative professions such as science and the arts (1–3), and the other way around: persons with creative occupations (2) and relatives of persons in creative occupations (4), have significantly increased risk of suffering from, amongst others, schizophrenia and bipolar disorder. Psychometric, clinical, neuroscientific and genetic research further suggests a correlation between (the likelihood of having) a bipolar or psychotic disorders and (heightened levels of) creativity or being involved in creative occupations (5–12). Although a correlation between the mental illness and creativity is plausible based on these studies, no final statements have been made about causality, leaving many questions open about the nature and direction of the presumed relationship.

In the history of thinking about creativity and mental illness, several theories have been proposed to explain the nature of the relationship. One that appeals most to the imagination is that certain aspects inherent to mental illness, may provide unique opportunities of inspiration and energy to create art (13, 14). This idea gained momentum within the anti-psychiatric movement of the 1960s and 1970s (15, 16), and was substantiated by the work of, amongst others, author and psychiatrist Kay Jamison (17) on bipolar disorder and the artistic temperament, but has also been criticized for its overly romantic view of creativity (18, 19). Another line of thought is captured in the “shared vulnerability model” (20, 21), which suggests shared personality “traits” between creative people and persons with mental illness, such as latent inhibition and hyperconnectivity. This hypothesis is endorsed by some empirical studies (22–26), but does not explain fully why mental illness is sometimes accompanied by creativity and sometimes not at all. Abraham (27) therefore suggested that the relationship may be best described as an inverted-U function of causality, to account for the conflicting findings, where mild symptoms of psychopathology may be beneficial, and severe levels of distress may only hinder creativity [see also (28)].

There are also authors who see no point at all in bringing the two concepts together and/or find it implausible that a relationship actually exists (29–31). They believe that findings suggesting causality are due to chance and are biased by persistent myths that exist in society. They argue that the most cited studies on creativity and mental illness have countless flaws and fundamental errors and some of them even question whether the subject can be studied at all, since it is almost impossible to come to consistent, consensual definitions and measures of the two major variables (32–36). Schlesinger (32) even warns that the tendency to investigate the link and make claims on basis of these studies has unfortunate implications for the perception of creativity (as people with exceptional gifts are pathologized), and the credibility of psychological research in general. Most authors agree, however, that despite the difficulty of investigating the relationship between creativity and mental illness, and the fact that adverse effects may occur, academic interest in this area should not come to a halt, but might call for other approaches (12, 37).

One approach that is eminently useful to move beyond the polarized debate of believers and non-believers into new research dimensions, is the qualitative approach. With the rise of the Recovery concept in mental health care, there is increasing attention for qualitative inquiry and the study of lived experiences of citizens and consumers. However, when it comes to the topics of creativity and mental illness, research focuses mainly on exploring how art practices can promote recovery and mental well-being (38–40), for example through the ability to narrate experiences (41). Art in this context is considered an intervention; a *means* to improve mental health. The broader and more open question of lived experiences with how creativity and mental illness might relate and interact (or do not relate and interact) can be considered equally relevant, but has thus far remained somewhat underexposed. One qualitative study, which analyzed experienced relations between creativity and bipolar disorder (42), suggests complex, reciprocal but also ambiguous relationships between mood and creative processes, where people narrated a positive impact of mood on creativity and vice versa, but also discussed the problematic sides of the same process. More qualitative research is needed to confirm and better understand these type of experiences, which is relevant to professional development in mental health care and (public) mental health policy and could also generate new research questions.

The present study aims to explore and analyze lived experiences of professional and semi-professional artists who have faced mental illness. The study focuses on how participants experience and understand the phenomena of creativity and mental illness, as well as how they perceive the relationships between the two in the context of their lives.

## Methods

### Design

A qualitative, explorative study within an interpretative paradigm, guided by hermeneutic phenomenological theory and methodology, as described by Dibley et al. (43). Hermeneutical-phenomenological thought (44, 45) assumes that there is no ‘single universal truth’ about phenomena. It focuses on the way in which individuals experience, describe, interpret and understand a particular phenomenon – in this case two phenomena and their mutual relations – and is characterized by an ongoing, non-linear, circular process of interpretation, guided not by fixed methodological steps. The researcher also brings his/her own being-in-the-world knowledge to co-construct, through language, with participants, a new interpretation of events (46, 47). The study was part of a larger qualitative study into creativity and mental illness, which explored perspectives of artists with mental illness, participants of community based art initiatives and professionals working in the field of art and mental health.

### Recruitment

For this study, professional and semi-professional artists in visual and performing arts, with self-reported experience in mental illness, were recruited. The first author searched online for (newspaper) articles, social media posts and websites about/by artists who are open about having mental problems. An informal letter was used to approach them (whether or not via their management) that contained information on the aim and method of the study as well as an introduction of the first author herself. Participants were also recruited through ‘Gallery Beeldend Gesproken,’ which is an organization that focuses on exhibiting, selling and lending art by professional makers with a mental illness story. A central contact person within the gallery mediated in the recruitment, by sending the informal letter to a selection of artists and passing on the names of interested parties (with permission) to the first author. If interested in participating, an official letter, including GDPR guidelines on confidentiality and data processing and the consent form, followed, and an interview appointment was scheduled. In line with hermeneutic thought, no fundamental exclusion criteria were applied. Florid symptoms of, for example, depression or psychosis, thus were not an exclusion criterion in this study.

### Participants and sample size

In order to achieve a high level of completeness, comprehensiveness and philosophical consistency (47), and to detect possible similarities (shared experiences), we recruited a fairly large sample size of 24 persons. Sufficient variation was sought in age, gender and art form. The authors feel confident that the final sample of 24 participants reveals the wide range and complexity of the experience of the relation between creativity and mental illness and is ample to answer our research questions (see Table 1).

**Table 1:** Pseudonyms, art discipline and mental illness experience of participants.

	Pseudonym	Art discipline	Self-reported main mental health issue / mental illness experience
1	William	Music	Bipolar disorder
2	Anne	Music	Depression
3	David	Theatre	Autism
4	Peter	Theatre	ADHD
5	Carolyn	Theatre	Anxiety or compulsive disorder
6	Dylan	Visual arts	Not specified
7	Matthew	Visual arts	Psychosis
8	Susan	Visual arts	Psychosis
9	Jeroen	Visual arts	Psychosis
10	Laura	Visual arts	Anxiety or compulsive disorder
11	Emma	Theatre	Eating disorder
12	Vincent	Music	Depression
13	Rose	Visual arts	Psychosis
14	Philip	Visual arts	Bipolar disorder
15	Michael	Music	Trauma
16	Jacqueline	Theatre	Dissociative disorder
17	Angela	Visual arts	Depression
18	Brenda	Visual arts	Psychosis
19	Scott	Visual arts	Anxiety or compulsive disorder
20	Andrea	Visual arts	Psychosis
21	Ruth	Visual arts	Psychosis
22	Lou	Visual arts	Autism
23	Karen	Visual arts	Bipolar disorder
24	Emily	Visual arts	Schizo-affective disorder

### Data collection

Single unstructured in-depth interviews, lasting an average of 72 min (range 42–144), were completed by the first author with each participant between August 2020 and February 2021. All interviews were recorded, transcribed verbatim by the first author, and pseudonymised. The researcher made field notes during and after each interview about her impressions of the interview, as well as during transcription. Due to the COVID-19 pandemic, the majority of interviews were conducted remotely through a video connection (except for 2 face to face and 1 by phone), but this seemed to enhance rather than diminish the ability to engage in deep conversation (48).

The purpose of the interview was to create an opening (‘clearing,’ 45-) that offered space for the participant to narrate his/her experiences with regard to creativity and mental illness and their (possible) relations. Each interview began with a more detailed explanation from the researcher about the research and her own relationship to the research topic. In order to connect optimally with the participant, no fixed topic list was used for the interview. Most interviews did however follow a similar structure, starting with talking about the participant’s art practice and his/her experiences with creativity since childhood, after which the subject of mental illness gradually got introduced, often by the participant as a natural part of the whole (life) story. The researcher used probing questions such as “Can you tell me what your art is about?,” “What do you feel when you are working on your art?,” “What does it mean to suffer from mental illness?,” to encourage reflection on a specific topic. The participant was in charge of telling his or her experiences; probing and follow up questions always matched the particular story of the participant. By actively and deep listening, the researcher helped the participant make his/her story coherent, for example by picking up things that (apparently) had meaning to him/her. Techniques of co-constitution were used during the interview to verify participant meaning and researcher understanding (49). “When you were telling about XX, I got the feeling that this relates to YYY, is that correct?.” Similarly, the researcher sometimes elaborated on notions raised in previous interviews: “Some respondents mentioned X as an important topic, how is that for you?.” In the last part of the interview, participants were explicitly asked to reflect on the public image of mental illness and creativity. The results of this part of the interview are beyond the scope of this article (which focuses on lived experiences and the self-perceived relations) and are analyzed and reported separately.

After the interview, a short questionnaire with general questions (both demographical and related to art discipline and mental illness experience) was administered. It was decided to complete the questionnaire afterwards, because the first author could then relate the questions to the story of the participant. This was especially important when asking about the nature of the mental illness. It was considered important to not ask participants beforehand what diagnosis they were given, to provide the space to tell an open story, not colored by medical frames.

### Ethical issues

Written informed consent was secured from participants for data collection. The right to withdraw was made explicit. Transcripts were pseudonymized and potentially identifiable data was removed from the transcripts to protect participants’ identity. The transcripts were stored in a secure location. Audio files were deleted after transcription. Recommendations of the ethics committee of the Trimbos-institute (registration number: 3028631/25-05-2020) to optimize procedures of secure processing of personal data, the provision of full and understandable (legal) information on study participation and assessment of the burden of the interview, were adopted. This non-invasive, qualitative study was judged exempt from a review by an external review board.

### Data analysis

As a guideline for the data analysis, we used elements of interpretative data analysis described by Dibley et al. [(43), p. 199]. The aim of the data analysis was to gain an understanding of the ways in which experiences of/with creativity and mental illness (may) cross paths or even merge in everyday life of artists-patients. Analysis was not aimed at finding ‘the truth’ about ‘the’ correlation between the two concepts, but to reveal perspectives on the issue of creativity and mental illness, and their possible relations, which may otherwise be hidden, through the telling of participants’ accounts of their experiences. The actual analysis phase began with carefully reading each transcript from start to finish, taking notes on salient elements in the text as well as the overall impression of the interview, after which initial codes were assigned to sections of text that seemed to be of interest. This lead to a first, still fairly global set of working themes per interview. A short, interpretive summary was written for each interview, based on these working themes, while staying close to the verbatim text. These summaries formed the starting point for a first attempt to identify similarities and connections between different transcripts. The second author was involved in this process, by critically questioning the steps that were taken and checking whether assumptions made were sufficiently substantiated by data. Mindmap techniques were used to facilitate this dialogue (50).

A process followed, which can best be described as a circular process of re-reading of the parts and the whole of the transcripts, interpretation, distilling (increasingly nuanced) themes, and coalescing similarities in common themes and shared practice. Software for qualitative analyses MAXQDA facilitated this process. Characteristic of this phase is the moving back and forth between emerging themes and patterns and the raw data, whereby re-reading the data can lead to enriching/deepening the themes or even making new themes emerge (43). With this process of ‘dwelling with the data’ and as the analysis phase proceeded, the first author increasingly recognized repeated presence/persistence of a particular theme or pattern, as identified in the interpretations, leading to a congruent interpretation of the data. Interim findings were critically discussed with the other authors on a regular basis.

### Reflexivity and rigor

The lead author, a female researcher in her 30s, had been professionally involved in applied mental health research for over a decade prior to this study, with a particular interest in the concepts of recovery and recovery-oriented practice. Her professional background in mental health care, including many interviews with people with mental illnesses, determined a large part of her prior knowledge of the phenomena studied. Being involved in art as an experienced amateur musician, further shaped her horizon of understanding on the topic being studied. Because of an interest in both the subject of mental illness and creativity, the lead author became fascinated by the cultural myths with regard to the mad genius narrative years before this study was conducted, and wondered how to value this romantic and fairly unilateral imagery. As she was convinced that both creativity and mental illness are not value-free concepts, always linked to experience, meaning and context, she got motivated to use an interpretative approach to the issue as a desirable addition to existing knowledge.

In qualitative research, and especially hermeneutics, it is assumed that ‘being inside and connected in some way to the phenomenon of interest, can bring huge benefits in terms of the ability to uncover meaning’ (43). However, under the condition that the effects of the positioned researcher, are taken into account (51). The main researcher kept a journal since day one, and throughout the whole study, to record and examine her own thoughts and preconceptions regarding the topics of creativity and mental illness. This journal was helpful, especially during the interaction with the data, to discover how her own perceptions relate to those of the participants, to put her own interpretations in brackets, and look more openly at the participants’ stories.

The pre-conceptions of the second author were formed by his academic background in medical humanities with specific focus on the way disease, including mental illness, is depicted in literature, and the third author brought along with him extensive experience in academic and applied research into community mental health care and recovery. The fact that the authors had different educational backgrounds (health sciences, medical humanities, social sciences), broadened the interpretation of the data. Reflecting on own assumptions and ideas about the relationship between creativity and mental illness, as well as those that prevail in society and are propagated by the media, was topic of ongoing conversation in the research team.

The authors further enhanced the trustworthiness of the study by pursuing robust, transparent description of research setting, participants, data collection, and analysis procedures, ‘rich, thick description’ (52) and a demonstration of the relationship of findings to the wider literature, as well as the use of verbatim quotes from participants to support findings being presented and the demonstration of shared as well as varied experience amongst participants. In the presentation of findings, in some quotes, questions or reactions of the first author are retained to make the course of the conversation clear to the reader.

## Results

### Participant characteristics

Study participants ranged from age 30–65 years (mean 41), and included 13 women, 10 men and 1 non-binary person. 62% of the study participants were involved in visual arts (painting, drawing, digital art, combined techniques), 38% in performing arts (music, theatre). 38% were involved in a second art discipline. 67% had received formal art education (on post-secondary vocational, bachelor or master level), 1 person could not finish it because of mental health problems, and 1 person followed a specific four-years art course for people with a disability. 25% were self-taught. 38% earned all of its income from art, 58% earned part of their income from art (in combination with income from other work or social benefits). 96% of the participants had past or current experience with mental health care. Their self-reported main mental health problems were psychosis-related (33%), mood-related (25%), anxiety-related (including obsessive-compulsive disorders and trauma) (17%) and ‘other’ mental health problems (21%), including eating disorders, autism, dissociative disorder and ADHD. One participant wanted to participate in the study, sharing his personal experiences on the topic, although he did not further specify his experience with mental illness and did not report past or current use of mental health services. 21% were no longer receiving any form of mental health care at the time of the interview.

### Qualitative findings

Analysis revealed three constitutive patterns describing self-perceived relations between creativity and mental illness in participants’ lives. However, before going into this and in order to properly understand these patterns, we first consider how participants describe and give meaning to the two phenomena (creativity and mental illness) separately.

#### The phenomenon of creativity

Participants understand the phenomenon of creativity as a constructive force of “making something that wasn’t there before.” However, more important than making something that is perfectly original, is making new connections in the pre-existing world and changing the course of something that seemed fixed. This process, also referred to as a process of problem solving, requires to be able to see things from multiple perspectives, and is not reserved for artists. On the contrary, participants regard creativity as a human condition. Although opportunities, circumstances, urgency and urge to develop one’s own creativity differ between people, participants feel that in essence there is a potential for creativity in every person.

Brenda: I actually understand creativity in terms of creative thinking. So solution-oriented thinking, thinking out of the box, very broad. And that can be very applicable in your whole life. If you have a technical problem, it is very different than if you have an emotional problem, for example, but they both ask for an answer or a solution or a creative interpretation to allow that process to continue. That's the gist of it for me.

Karen: You can look at a thing from one side, but if you're creative, you can see it from at least 50 sides. That is creativity across the board. I think that everyone has creativity and that you can nurture it but you can also destroy it. (..) I think if you're encouraged to fantasize and play as a kid, creativity is nurtured, and if you're brought up in a very practical way or if people are being negative about what you're doing, you can break that early on.

Zooming in onto the phenomenon of *artistic* creativity, participants describe this as a process of making the unconscious conscious, or giving a meaningful shape to a specific content, often related to something the maker is concerned with: a personal feeling, a thought or “something that is going on in the world” that he or she wants to understand or address. Depending on the aim of a creative process (autonomous, social, commercial), there is more or less of the maker’s subjectivity contained in a work. However, where the maker has completely disappeared from the work, “it can no longer be called art,” participants say. Participants underline that a certain level of craftmanship with regard to the specific art discipline is necessary to make valuable work. This does, however, not necessarily have to be obtained from formal training; self-thought artists may even have “an edge” in expressing themselves artistically. More decisive than technical skill is the way an artist is able to engage himself in the creative process.

Philip: Artistic creativity for me stands for making visible, making tangible, a very personal, inspired thought. That's it.

Jeroen: So an artist notices a collective tension that generally goes unnoticed, that's how it feels to me. This tension is just hanging in the air, if you can tap out of that, then you're a real artist. This is not dependent on whether you are formally trained. If you are an artist, you bring something that the whole world is unconsciously involved in, as an artist you make it conscious, through you.

When reflecting on their own creativity, participants describe an active process, where they enter into a relation with the work that is created, and with the future consumer of the work. Participants clearly indicate that being able to do this, is not something that comes from a personal ‘source of talent.’ When participants speak of talent, this is at most the capacity that one “apparently has” to generate a multitude of ideas, to do something with those ideas, or the ability to commit oneself to the (hard work that) the creative process (requires). The same applies to the notion of ‘inspiration.’ Having inspiration is not perceived as an exceptional merit or special quality of their own. Inspiration is experienced as something that comes from a “natural tendency to constantly process information” and “actively make connections,” which is just something they do and have always done. The artistic engagement in ideas that impose themselves on participants is experienced as self-evident and even has an almost involuntary nature (see also Flow).

Emma: People often think it is a passive process of having talent and inspiration, that *that* is creativity, while I see it as an active process, just like in sports or research. You engage with it. You enter into a relationship with it.

William: People sometimes say ‘do you have enough inspiration?,’ like it is something I have to wait for. I always find that a very strange question. I have so many ideas that I hope I live long enough to be able to execute them all. So that's kind of a Bulimia-type problem: the longer I live, the more ideas I get, so to speak. Inspiration or creativity is more a matter of selection what to tackle. The core is a kind of multitude of voices and ideas, that is just there, shall I say. And in that multitude, there is also the future audience, ‘Who am I going to make that for?’.

#### The phenomenon of mental illness

When describing the phenomenon of mental illness, participants tend not to separate the issues of ‘what it is,’ ‘what it does,’ and ‘what it means to them,’ which is reflected in the use of terminology. Although most participants do not mind using terms such as ‘mental disorder’ or ‘psychiatric problem’ because “that is simply the word most often used for it,” most participants prefer other terms, including mental/psychological vulnerability, − limitation, − handicap, − struggle, − fear, − pain, − dislocation or – suffering. Terms with a more positive connotation are also used, such as: neurodivergence, self-exploration, challenge or sensitivity. Such terms reflect the fact that most participants do not necessarily regard what they experience as pathological. Almost on the contrary, most participants see mental struggles, vulnerability and suffering as something that affects every person and takes different forms depending on the person. They do believe that the degree of struggle and suffering and the extent to which this affects life can differ from person to person. Participants feel that what they are experiencing/have experienced is likely to be more intense, longer-lasting and more disruptive than for people who are not labeled as ‘mentally ill.’

Rose: I don't think I'm ‘ill’…. Doesn't really fit somehow. While of course you can suffer really extensively. But I'd rather call that disorganized, upset.

Peter: For me it's actually an investigation, that's the best word. Someone had also given me books about it and then I thought: I don't really care what the label is, I find it much more interesting that I start to accept that my brain works the way it works. And that is now starting to find its way. ‘Ok so I function like this, and how can I relate that to that.’

When participants describe the *experience* of living with a mental illness, beyond definition and terminology, they narrate (a continuous threat of) a psychological and existential process of alienation, characterized by loss of control and connection with one’s own subjectivity, with others and with life in general. This loss of connection is often triggered by internal processes (speeding up or slowing down of thinking, feeling, for whatever reason) or external processes (demands from the outside world that the individual cannot/should not meet). In “bad periods,” participants experience feelings of worthlessness, fear, self-doubt, shame, disinhibition or confusion. These are initially mild, but gradually those feelings take up more and more mental space, at the expense of the personal sense of self, which is slowly repressed and sometimes even seems to disappear completely. “That is when I lose myself.” This experience is, however, not only an internal one, it also separates a person from the outside world, deprives him of the opportunity to take a place there and isolates him, which is, of course, reinforced by the way the outside world cuts ties with those whose mental suffering is no longer within accepted limits. It is this disruption from ‘normal,’ daily routines and participation in social roles, that participants call a an actual ‘crisis,’ often described in metaphors such as: “a radio going to noise,” “entering a void,” “entering a silo,” “meeting the monster that shuts down my life again.” This seems to always come with a sense of failure, which makes the experience even more distressing. For most participants, periods of crisis alternate with periods of relative stability and recovery of social roles, whereby the experience of suffering itself fades away and “only the story of it remains.” However, for a small proportion of participants, there is a constant feeling of confusion and alienation, often since childhood.

Anne: It basically cuts off any connection to yourself and to the outside world. In a very rigorous way. It feels like a kind of alienation from… from everything. And the beauty of the human mind is that the moment you are not in there, you are also able to forget how it feels exactly. Rationally I know how it is, I can look at it, I can describe it, but really being in the middle of it, that's something you forget. You have to.

Lou: I experience a gap between me and the rest of the world. Gap does sound a bit ….. Yes. Well at least I don't … mmmm … I don’t know well how to move in the daily reality, as I see other people doing. (..) In the end, that's … it's always really gotten in my way. I thought I wasn't trying hard enough to be a full-fledged social participant. Yes, I've been confused about that on a regular basis, just to make an understatement.

One aspect that most participants mention when describing the phenomenon of mental illness, is that although it is accompanied with involuntary and threatening experiences, it is at the same time not seen as something exclusively negative. The paradoxical truth for participants is that mental illness always entails the need for refutation. Mental illness entails the project of learning how to respond to and understand your suffering which for them often leads to “a deeper understanding of existence” in all its dimensions and contrasts. This heightened understanding of life is why many see mental illness as “something that can add value.” Although the social and cultural narrative about mental illness is predominantly negative, participants see this as a reduction in relation to how they experience it.

Laura: I've wasted ten years of my life. I've always said I'd like to turn that back, but no, because it also taught me so much, about myself and about working with others, about asking for help isn't a bad thing, crying isn't a bad thing, you know, you learn so much from it too. Of course you keep thinking occasionally: what would I have done differently? But on the other hand, you know: it happened, and what I got out of there is so beautiful … JB: Can you explain what makes that beautiful? Laura: I think wiser is the right word, because you get to know yourself so well. And to get to know yourself, even the less pleasant things, that is such a revelation (..) that brings you so many beautiful things.

JB: And you could even say, I say it very carefully, but is there also beauty in suffering? Carolyn: ….. Well there's beauty in self-examination I guess. There is beauty in self-examination and examining other people's motives. You get the need for it when you suffer. So there no beauty in suffering but beauty does come from suffering I would say.

#### Self-perceived relations between creativity and mental illness

Creativity and mental illness emerge in the interviews as phenomena to which participants actively relate themselves. Participants use the phenomena to understand themselves and explain their live courses. Both phenomena are inextricably linked to their identity. But it does not end there. An overarching theme in the interviews is that the two phenomena influence each other in various ways. Three constitutive patterns that describe the self-perceived relations between creativity and mental illness in participant’s lives, are: “flow as a powerful force”; “ambiguous self-manifestation”; and “narrating experiences of suffering.” These constitutive patterns were informed by 12 relational themes (Table 2). Below these patterns are elaborated.

**Table 2:** Constitutive and relational patterns and codes.

Constitutive pattern	Relational patterns	Codes
*Flow as a powerful force*	Acceleration	sucking up information; idea generation; inspiration; dedication; letting go of rationality; creative process and mania; creative process and voices
	Transcendence	meditation/mindfulness; relaxation; elation / joy / euphoria; distraction; playfulness
	Overload	overload; engulfment; exhaustion; provoking / incitement of symptoms; crossing borders; methods of slowing down
*Ambiguous self-manifestation*	Statement of existence	being there in the world; being seen by others
	Coping	creativity as coping mechanism; creativity as escape route; creativity as counterbalance
	Recovery	to come in action; processing emotions; fire / life direction; taking up space; help someone else; role recovery; to connect; making story coherent
	Struggle	attack of self; self-doubt; ambiguity toward the made
	Stagnation	mental illness as obstacle to creativity
*Narrating experiences of suffering*	Communication	language; expression; parallel world; taboo subjects
	Unconscious storytelling	symbols of suffering; color and shapes
	Conscious storytelling	mental illness as inspiration; mental illness as theme; communicate suffering; helping others; being vulnerable, distance from story
	Voice & connection	solidarity/connection; universality, facilitating dialogue on suffering; give suffering a voice; taboo breaking.

##### Flow as a powerful force

Making art in a broad sense admittedly may involve planning, thoughtfulness and conceptual reflection. However, the actual process of making things come into being, is experienced by participants as a largely intuitive process that has some elusive and “mad” sides to it. Exactly what happens there, is difficult to put in words, participants explain. Participants describe an acceleration, an increasing surrender to the ‘thing being created,’ resulting in a flow or hyper concentration, a movement toward a place “where time no longer matters” and where the final result stays uncertain until the process is complete. Participants suspect that their mental illness makes it easier for them to enter a state of flow than others.

Karen: I'm just making, I don't think at all, I don't have a plan at all. (..) I just start and then I see where it ends. Yes, when I'm drawing, I'm completely involved in that process, I'm only concerned with colour and shape and what's on paper evokes the following. It is as if you surrender to the process. Not thinking at all.

Dylan: Eventually, until I'm done, I don't understand the whole story of what I was making. But then everything seems to fall into place at once, like 'hey how can that be?'

The flow participants get into while creating, is described as a state of mind with therapeutic potential, mainly due to the fact that flow takes participants away from the cognitive, the cerebral, and “opens the door to a different way of experiencing the mind,” a more direct experience, without the intervention of thought or conviction. Flow therefore becomes a “highly meditative experience” that brings a sense of elation, relaxation, playfulness or even euphoria. This results in a distraction from negative thoughts and feelings, it helps to create “space in your head” and eases mental pain. For some it represents a “safe world” to escape to. Even experiences that are disturbing in another contexts, such as hearing voices or seeing images, can be experienced in a positive light, because as part with the flow, they become one with the artistic process.

Michael: While playing the piano then you don't think about anything, I don't think about anything when I'm playing. Great, then you don't have to think about anything bad either. Yes, actually I've always used that as a numbing agent, or as an emollient.

Susan: I'll show you, look, this kind of work I make if I'm really totally in a flow ….Yes then it's like a playground, then I'm talking to myself, I'm listening to music, and everything flows and everything swirls. There's no inhibition and no … it's just completely … totally loose! And there is also a voice that facilitates that process. She is present during such a making process and in my opinion she also regularly ends up on the paper. Kind of a fabulous creature it is, it's half human, half animal, and sometimes I think she's an angel you know, she interferes with me regularly so. (..) She very often helps me to get into my work process.

There is, however, also another side to the experience of flow. Flow can become ‘too much.’ It can become an engulfment, with the artistic process gradually taking over and crowding out other aspects of life, such as family and household chores. It can become a kind of “egocentric process” in which everything outside of that process loses relevance. More dangerous is that it can jeopardize self-care. For example, participants describe staying up at night to work, or stop eating, when the flow takes over, with possible consequences for exhaustion and worsening of or relapse in psychological symptoms.

Laura: That trance you enter, that can of course also make you forget that you still had to vacuum, and that you still had to do your dishes or whatever, because then your partner comes home and then there is still food on the table from this morning or something. So yes there are dark sides to it. I can get into it too much. It's very egocentric. (..) because when I do that, I really only spend time on myself and then I figuratively shit on everything around me, everything just has to wait.

Angela: I can work for a very long time. I work nonstop then, which is really kind of manic. I also have those hypos that I go on endlessly and do not eat. And especially now that my kids are grown up, it's a lot easier to forget to stop, because you can leave everything behind, that is really dangerous.

Since flow has both constructive and destructive potential, all participants face the challenge of learning to manage it in such a way that they can be carried away with it, but not too far. Often this includes a conflict between artistic interests and health interests. Usually the artistic process requires just a little more ‘flow’ than is healthy for the body and mind. Participants describe a search for boundaries, a pushing of boundaries, in order to make art, while trying to avoid ending up in a mental crisis that stops the artistic process altogether anyway. Many participants, especially those who have other obligations in addition to making art, such as children to care for or other work, quite rigorously restrict their flow, for example by setting strict rules about how long they can create, by working with others or by taking medication. They ensure as it were, that ‘healthy flow’ is separated from ‘potentially problematic’ flow. At least, they try; learning to put brakes on it is considered a permanent challenge by many.

Scott: I do use more and more medication to keep it under control. About a year ago, then it really started to get a little out of hand, I kept working, couldn't sleep, I couldn't eat and all that. I wasn't in this world at all anymore actually. It got really too exhausting you know. So I decided to increase my dose a bit. I'm a bit dulled now of course, but I notice that the creative process I just talked about is still going on.

Susan: In my case I think the balance is important. I'm very creative, so I have a lot of associations, a lot of connections I make all the time. You know? And if I'm positively busy then that has added value, certainly in my work, but if I overdo it, then I can't do my work anymore. So that's a precarious balance, where you try to balance between indeed going too far into your own psyche, or using it for the greater good of creativity.

For some participants the dilemma of slowing down/ putting a brake on the process is less evident. They have arranged their lives in such a way that they can afford to lose themselves more in their art-making practice. Often they live alone, work alone and spent almost all their time with their art.

Andrea: I told that psychiatrist about that voice, that psychiatrist said I have to take pills haha. And I brought those pills home, but it was really a choice, should I go on like a leaf or a human, okay that's hard, because then I would have nobody to help me, but I decided to go through it without medicines. And because of that I really have to keep working alone because I have to live in isolation, as a hermit, yes that social life for me does not exist. That's okay. When I was young I did it a little bit. But not anymore after that. Only good feeling I have is when I draw. So I can't do anything else.

##### Ambiguous self-manifestation

The second pattern that links experiences of creativity and mental illness in the lives of participants has to do with the existential opportunity provided to the maker to manifest (parts of) himself through his art. Many participants experience making art as a way of creating or *recreating* themselves and some even suspect that an ‘incomplete self’ is the whole reason for wanting to make anything at all. Because “every work of art is absorbed” in the maker’s sense of self, to a greater or lesser extent, and parts of the maker’s sense of self always “end up in the work,” a continuous loop of self-examination and self-affirmation is created.

William: Or you can put it another way: I don't know exactly who I am and I have to figure that out every day. I have to rethink who I am every time. But you can also say it is not certain whether I have an already defined ‘self,’ and that my ‘self’ must always be raised from what I make. It's a kind of structure that gets built every time and is gone the next day, as it were. Like a sand castle. (..) There's something very existential about it.

Dylan: Every work is the rebirth after the completion of your previous being. Do you understand? You are no longer what you used to be. And so I keep making a new ‘me’ every time. JB: That does mean that you and the work are one? Dylan: *Were* one. Yes. If you see my painting then you can see my old self. JB: Your old ‘me.’ So you build yourself on your old selves, through your art? Dylan: Yes, of course. (..) It's kind of a mass grave. Just stack. Just based on my old selves.

The process of self-manifestation through art is reinforced when the created comes into contact with the outside world. At that moment, part of the maker’s ‘being’ is revealed to others. Making yourself known to others through art, “where there is the possibility of others to respond to it” can be a strong form of connection with the outside world and a statement of existence. Making art, with that, has for many participants becomes a constructive response to the alienating destructiveness of their mental illness.

Emma: And well, an eating disorder like this, and that will undoubtedly apply to other psychological struggles, is also a search for who you are and what place you can take. How I experience it is that that creativity, or that makership, that also gives me a feeling of existence, a ‘me,’ an identity. If that piece grows, then the other [eating disorder] part shrinks. That is literally how I once drew it in a research for one of my performances, yes. I really believe that. One grows and the other shrinks.

Jeroen: The psychological need creates necessity and necessity creates a solution. Creating is just connecting, associating, bringing things together. It is actively creating yourself again. Out of necessity, because something is broken. When you are whole, you do not have to create anything anymore.

However, participants also indicate that it does not always work that way. The fact that one manifests oneself in the creative process can just as much be the cause of more (self) doubt and confusion, for example when makers do not feel connected anymore to the things they made, or see things of themselves reflected in their art that frighten or worry them. In the worst case, the process of self-manifestation can amplify the (self) destructive effects of mental illness, instead of leading to some kind of relief.

Anne: Art making is entering a vessel of connections. Indeed, I think that connection, is the negative of the picture which is depression. However, ultimately it doesn't help. Nothing helps. That's very stupid. It doesn’t help. Reading or listening back your own work even makes it worse. Often the connection with what you have made is gone. So it doesn’t help, rather, it induces more fear because you think ‘hey I don't feel this at all anymore,’ ‘I don't understand why I made this,’ ‘I don't see the point in it.’ You know. Apparently I felt the point at the time and that discrepancy is just very frightening.

Scott: It's very contradictory actually. Apparently it is deep in the human being that you want to exist and be seen or something. I do want to be in the picture … I want to exhibit my work, and that also helps me. But sometimes, it can also be halfway through an exhibition … then it hangs there and suddenly I get a kind of panic, you know, then I actually want to burn everything and I want it to be erased, you know, that I never existed, preferably actually. (..) But that’s not possible. As an artist you are actually leaving a kind of trail behind.

The fact that making art can, in addition to being a counterweight, also confirm feelings of alienation, which can even bring the creative process to a complete standstill, does not prevent artists from returning to art again and again. The urgency to make art ultimately remains: “art always wins.” Participants simply cannot imagine they will ever stop making art; they accept that it entails a struggle with themselves from time to time, a tension that is always on the lurk, unavoidable in essence, but perhaps what makes their work interesting.

Angela: I have a very strict ‘me’ that says: it makes no sense to make that art because it won't work anyway, it's nothing, stop it, just go and find a job for four days a week and that art that makes no sense. And that's that nihilism, it is very difficult. And yet ….yes every time … I am so very quickly caught by … when I see shapes. Abstract shapes.. Then I think oh wow! So the urge to go to work eventually overcomes. Yes the senseless is repressed. Yes then I just get going instead of ….I really taught myself that, without hurting myself, without drinking, drugs I certainly don't do that anymore, but to choose: sit down, and start working. But that nihilistic feeling, it will always be a part of me and I think it's also visible in the black and white and colour of my work. The tension will never be solved, but I cherish the art that comes out of it. It produces a beautiful picture.

##### Narrating experiences of suffering

The third pattern found in the data revolves around the content of the work and the function that art can take on as a bearer of *stories* on issues that are precisely difficult to express through other means, including experiences with mental struggles and psychological suffering. While those kind of experiences are often “difficult to talk about in everyday life,” because they make others uncomfortable, art can be a means of consolidating and communicating about it, albeit in a roundabout way. Art is a kind of language, many participants say, whereby feelings and thoughts that live in the inner world are given a solid (or fluid) shape outside of the mind, and in that capacity can be transferred to others, who might recognize these feelings and thoughts consciously or unconsciously, which in turn creates a joint story between maker and consumer of the art work.

Anne: When you're depressed, all people want to hear is ‘how are you feeling,’ which is almost indescribable, plus it's a very boring story. Same story every day. And people don't want to hear that, people want to hear that things went a little better than the day before. And the moment that is not the story you can tell, then art is indeed all that remains because in art it is about the form in which you cast it. So when words are not enough anymore, the verbal, the ordinary communicating with people, then you still have a means of communication left, and that is the art. The way it comes out is very different, but forming ideas about that meaninglessness, that's a conversation that still continues, so to speak.

Usually, participants do not intentionally put stories about psychological suffering in their art. Yet they, retrospectively, see their experiences reflected in their work, sometimes literally, sometimes symbolically, for example in form, use of color, atmosphere or in the way in which certain themes, scenes or characters are portrayed. Dark stories of confusion, desertion and meaninglessness can be hidden in the work, as well as stories, which are precisely the opposite: stories about a world without suffering, a world of harmony, lightness, beauty, childlikeness and peace.

Scott: (..) It is kind of an obsessive story when I look back at it now. Often when you have exhibited something, then a distance actually arises. You automatically get a kind of retrospect. Then I have to admit that I also see something of the fact that I was really in a psychotic state when I made it. I think that is visible, afterwards.

Andrea: My art is about the inner, feminine paradise. haha. My personal life hasn't always been great, (..) but you know I'm trying to find a way to get around it, I think. I fantasize about life: what do I want to feel happy? So that's a kind of medicine too, all those painful experiences in my life, I process them, in the opposite form. (..) On the canvas I can do everything, I can tell the story of how life should be. So flowers, beauty, youth, everyone understands each other. Everything I didn't get in life.

There are also participants for whom the narrative potential of art is used more consciously and purposefully. For example, they literally depict the hallucinations they see, or use their experiences with depression or anxiety as themes in their work, often with the explicit intention of publicly exploring these experiences, engaging in dialogue about them and “helping others realize that they are not alone.” Portraying and revealing one’s own experiences in this way is described as quite difficult, exhausting and sometimes confrontational. Participants point out that it can make you feel vulnerable and that you need to be able to create a certain distance from the story you are telling. But it does open up a way of communication that contributes to a greater goal. These participants are aware that their story of mental struggle or suffering through art can be a powerful tool for connection, solidarity and togetherness among their audience. With their art they contribute to the proverbial ‘space’ in society where suffering is allowed to exist, and where people can come together to experience this space through art. In doing so, they also provide a counterbalance to the dominant space in which suffering has no voice.

David: Yes, because you have to process the stories you tell first. The stories I'm telling now I wouldn't have dared to tell in my first show. Because I've only just processed them now. (..) I'm also talking about things I used to be ashamed of. And now I try to see the power of that. Because it also just makes me unique, no matter how cliche that may sound.

Vincent: I notice that my music, the combination of the subject and maybe how I bring it or whatever, that really appeals to people, and I think that's very special, that I now have a kind of fan base of people who really know exactly what I experienced, say because they have experienced it themselves, and that's why it feels also as more than making music, it is like sharing or discussing something with people, and I also use my social media a lot for that to give space to say the struggles of other people. So in that I feel like I'm trying to do something more social or de-stigmatizing than if I wanted to be a pop star or something, that's not my goal.

## Discussion

This study is based on in-depth, open interviews with 24 professional and semi-professional artists in visual and performing arts who have experience with mental illness. The qualitative results provide insight into how the phenomena of ‘creativity’ and ‘mental illness’ are experienced and given meaning by participants and how they perceive their mutual relations, in the context of their lives. Findings reveal that professional artists with mental illness perceive many different relations between experiences of creativity and mental illness in their own lives. The two phenomena regularly touch, influence, nourish, undermine and reinforce each other in different ways. The findings do not suggest an unequivocal relationship, rather they reveal a whole range of complex, layered and ambiguous interactions, reflected in the creative process itself (flow), in the interactions between maker and the created (self-manifestation) and in the potential of art as a way of storytelling (narration). Through all these patterns, it is palpable that the experienced relationship between creativity and mental illness is full of contradictions and tensions: symptoms of mental illness can accelerate creative processes, but also inhibit them; feeling involved in a creative process can be a mindful and self-affirming activity, strengthening sense of identity, but it can also exacerbate suffering and confirm self-doubt; art can be a powerful tool to break taboos, but displaying mental struggle in art, means feeling vulnerable. All this leads us to the conclusion that while a relation between the two phenomena, in the experience of artists with mental illness, is confirmed, simplified ideas about this relationship fall short. In follow-up research, the opposing movements in- and the specific mechanics of the relationship between creativity and mental illness should be further explored.

In our findings we encounter many ideas that have previously been suggested by other authors, amongst others the idea that there may be creative potential in mental illness (8, 11, 53) and that there indeed seem to be some similarities between the creative mind and states of mental illness, including elements such as information filtering issues, curiosity and hyperconnectivity [(7), see also (54)]. Especially the findings with regard to ‘flow’ appeal to these previous works and are reminiscent of Jamison’s work (17) in which mood changes may nourish intense creative states marked by an expanding mind, a flood of thoughts, a heightened ability to generate ideas and make new connections. This does mean that the same criticism leveled at Jamison (18) could also apply here: the artists in our study may have been influenced by cultural images of the mad genius, which automatically places experiences with creativity and mental illness in a romantic framework, suggesting that mental illness can lead to artistic ‘highs.’ In the same way, common critique about the sample method which selected artists with mental illness experience, that were eager to talk about the mutual relations between creativity and mental illness, and gave no attention to creative people who manage to be both prolific and stable (55), applies here. Although both strands of critique are valid and need to be kept in mind when interpreting the results, there seems to be enough reasons not to dismiss the idea of ‘flow’ as a central concept linking creativity and mental illness solely as a romantic cliché. Especially since the finding that the process of flow can be both a pleasant, even therapeutic *and* a frightening and disruptive experience, poses an urgent self-management challenge, at least for the participants in our study. It is this contradiction that calls for more thorough investigation to be able to understand what is actually going on the lives of artists with mental issues, and perhaps find clues on how to support them better.

Another relevant finding in our study, which requires further investigation, is the opportunity that art offers participants to manifest themselves and thereby respond to the destructive aspects of mental illness, but where the opposite effect (an increase in symptoms) can also occur. In previous, mostly qualitative, studies it was shown how mental health consumers can benefit from art activities to enhance their process of personal recovery (56–62). Mechanisms of empowerment, motivation, confidence, self-expression and connectedness, all seemed to underlie the positive effects of art. This finding is in line with Taylor et al. (42) who conclude that creating/ making art can make you feel “like you are you” again, which can help in coping with mental symptoms and may even enhance the process of (re) validation of the self. In our study, professional and semi-professional artists also appeared to be looking for answers to the destructiveness of mental illness and opportunities for personal recovery, although the role of art in this was not unambiguously positive. Art making could be a firm statement of existence but also easily lead to more confusion or fear, especially when participants felt alienated to the work of art they made. This finding was not seen in the studies on mental health consumers, suggesting that there are differences between professional and leisure forms of creativity, in the extent to which it can be helpful in recovery from mental illness. It would be worthwhile to study what is behind this observation.

Thirdly, our study shows that artists with mental illness use their art to unconsciously or purposefully tell stories about psychological suffering, and that this also has a communicative and social component or purpose. It is known that art can contribute to reducing taboos and stigma regarding psychiatric disorders [see f.e. (63–66)], but here too, the research has mainly focused on the participation of consumers in arts practices, where the exhibition and appreciation of the previously hidden talents of the participants provides an important impetus for adjusting the one-sided image that prevails of people with mental disorders. The nuance that emerges in our study is that for professional artists, the communicative and social aspect of their art is less about reducing taboos and stigma surrounding specific conditions, and more about creating space in the community in which suffering (in its more universal dimension) is allowed to exist, and bringing together and connecting everyone who feels addressed by this theme, which is depicted in their work of art. It thus concerns a different way of influencing social structures, of which taboo reduction is a possible consequence but never the primary goal. This finding gives reason to focus future research on the role of art as a connecting mechanism in society, especially in times of increasing rapid social change and societal uncertainty, and to further investigate how art can contribute to public health in this way (67).

While strengths of our study include the relevance, the large sample size, the open interviewing, the thorough analysis and the choice to let participants define the core concepts themselves (instead of using predetermined definitions), the study also has several limitations. The first being related to the uncertainties about characteristics of the sample, which raises questions about who and what the findings actually relate to. The findings describe how persons with self-reported experiences with both mental illness and professional art making experience, reflect on the relationships between mental illness and creativity in the context of their daily lives. The recruitment strategy resulted in a very diverse group of participants, and no measures were taken to map the extent, seriousness and nature of the mental illness, nor to confirm creative aptitude. This was intended and in line with the methodological stance (43), but does influence transferability. The findings should not be seen as defined answers to the question of how the relationship between mental illness and creativity is perceived, or as applicable to every visual/performing artist with mental illness. Rather, they should be seen as context-related reflections that we have synthesized to open new ways of understanding of the subject. Future research could build on these and other findings to define new questions in more specific contexts and different settings, with which insights can be gained that are important for determining the role of art within public mental health and mental health care. Comparing findings regarding different types of mental illness experiences (psychosis, depression, anxiety) and different art forms (including art forms that were omitted in this study, such as literature, poetry, digital art and dance) should be part of this research.

Second, as is the case in all qualitative (and probably also quantitative) research, the position of the researcher influenced the way the data was collected and interpreted. Following Malterud (51) the question is however not whether the researcher influences the process, nor whether such an effect can be prevented. “This methodological point is transformed into a commitment to reflexivity, which means recognizing that knowledge is partial and situated, and to adequately take into account the effects of the positioned researcher.” In our study it was attempted to adequately take these kind of effects into account by reflecting on the role of the researcher and her personal and professional background, motivation and theoretical assumptions, by keeping a reflective diary, and by adhering to methodological standards. We believe, with Malterud, that preconceptions are not the same as bias, unless the researcher fails to mention them. A final limitation that must be mentioned is that, with the one-off, individual interviews, little insight has been gained into the context and daily practice in which experiences with creativity and mental illness come together. The data is actually based on a series of “snapshots,” and also relies heavily on the participant’s reflective abilities. It would be very interesting in further research on this topic to include other qualitative methods, such as participant observation, focus groups, or multiple interview series, to get a better idea of what the interactions look like in daily practice. A deeper analysis of the data, based on hermeneutical literature, would also be relevant. The opportunities of analysis through the phenomenological hermeneutical body of thought were only partly, and not fully, used.

Despite the limitations, we hope that this study contributes to the understanding of the relationship between creativity and mental illness. It should be noted again that the aim of the study was not to endorse (or falsify) the existence of a fundamental correlation or causal relationship, but to add perspectives to the academic debate that has been polarized into believers and non-believers. It is clear that many artists do not have mental illnesses and many people with mental illnesses do not have artistic talents. Myths about ‘mental illness as the path to (exceptional) creativity’ and ‘madness as a prerequisite for artistry’ must be seen as an overly reductionist and linear representation of the relationship [see also (42, 68–71)]. The same goes for views of the relationship that are locked into an intervention paradigm, reducing art to merely a means to achieve improvements in mental health. Our findings underline that none of these ideas seem to do justice to the whole story, as their opposing forms also emerged in the participants’ stories, and the large group of artists and patients who do not experience a relationship at all, were not interviewed. However, this does not alter the relevance of further and broader exploration on interesting leads, such as the opposing forces in the making process, the differences between professional and leisure art practices in relation to recovery, and the opportunity for creating space within which suffering can be accepted and ‘borne’ together, without further problematizing or medicalizing it.

### Practical implications

Our study confirms that although participants do not glorify the nature and impact of their mental struggles, are well aware of its destructiveness, and also seek or have sought mental health care, the experience of mental illness is not unequivocally negative. It can also open a way to other forms/levels of consciousness and (creative) dimensions of life that would otherwise have remained shrouded in darkness. At the same time, the study challenges simplified or romanticized conceptualizations of the relationship between creativity and mental illness, rather suggesting a whole range of interactions with different meanings. Understanding artists’ experiences with mental illness can help shape the role of art in public mental health and mental health care in ways that transcend instrumental and clichéd views of the relationship.

## Data availability statement

The datasets presented in this article are not readily available because the dataset consists of rich and complex qualitative data that is difficult to anonymize without losing crucial information. Data can be requested from the lead author, but any portions that may contain identifiable information will be removed. Requests to access the datasets should be directed to Jboumans@trimbos.nl.

## Ethics statement

This study was reviewed and approved by the ethics committee of the Trimbos-institute (registration number: 3028631/25-05-2020). All procedures were in accordance with the local legislation and institutional requirements, and with the 1964 Helsinki Declaration and its later amendments or comparable ethical standards. This non-invasive, qualitative study was judged exempt from a review by an external medical-ethical review board because it concerns a non-invasive, qualitative study. The participants provided their written informed consent to participate in this study.

## Author contributions

JB: Conceptualization, Formal analysis, Investigation, Writing – original draft, Methodology. AO: Conceptualization, Funding acquisition, Methodology, Supervision, Validation, Writing – review & editing. HK: Project administration, Supervision, Writing – review & editing.
